# Risk reduction after bicycle, scooter, and skateboard-related head injuries through helmet use and brain injury education: A trauma center quality improvement initiative

**DOI:** 10.1016/j.bas.2025.105858

**Published:** 2025-10-28

**Authors:** Leila L. Etemad, Lawrence Chyall, Sara Cole, Cathra Halabi, Gabriela G. Satris, Christine J. Gotthardt, Joye X. Tracey, Kathryn S. Park, Theodore T. Tran, Diego Martell, Bukre C. Coskun, Allen Y. Fu, Mahmoud M. Elguindy, Maria C. Velasco, Anthony M. DiGiorgio, Phiroz E. Tarapore, Michael C. Huang, Geoffrey T. Manley, John K. Yue

**Affiliations:** a Department of Neurological Surgery, University of California, San Francisco, San Francisco, CA, United States of America; b Brain and Spinal Injury Center, Zuckerberg San Francisco General Hospital and Trauma Center, San Francisco, CA, United States of America; c Weill Institute for Neurosciences, University of California, San Francisco, San Francisco, CA, United States of America; d School of Medicine, Medical College of Wisconsin, Milwaukee, WI, United States of America; e Department of Neurology, University of California, San Francisco, San Francisco, CA, United States of America; f New York University Grossman School of Medicine, New York, NY, United States of America; g Philip R. Lee Institute for Health Policy Studies, University of California, San Francisco, San Francisco, CA, United States of America; h Department of Neurological Surgery, San Francisco Veterans Affairs Medical Center, San Francisco, CA, United States of America

**Keywords:** Closed head injury, Helmet, Injury prevention, Quality improvement, Traumatic brain injury

## Abstract

**Introduction:**

Helmets reduce head injury severity after bicycle, scooter, and skateboard injuries. Prevention of one head injury reduces lifetime risk of reinjuries. Our clinical care quality improvement initiative (QII) aimed to improve helmet usage, education, and safety awareness in head injury patients at a United States trauma center.

**Research question:**

To assess QII feasibility.

**Material and methods:**

Head injury patients presenting to emergency department (ED) after bicycle, scooter, and skateboard accidents without helmets or with lost/damaged helmets were provided helmets free-of-charge, and in-person review of traumatic brain injury education, resources, and follow-up care. Surveys on helmet use were conducted in the ED and at ≥1 telephone appointments (2-weeks to 1-year).

**Results:**

In 21 patients aged 37.7 ± 12.5-years, 71 % were male, 38 % had traumatic intracranial hemorrhage on head computed tomography (CT) scan, and 81 % were unhelmeted. Mechanisms included scooter (48 %), bicycle (38 %), and skateboard-related (14 %) injuries. All patients reported improved understanding of risk reduction strategies and helmet use at enrollment and follow-up. At follow-up, 11/21 patients were able to resume pre-injury bicycle, scooter, and/or skateboard-related activities, of which 82 % reported consistent helmet use. Of 10 patients unable to resume pre-injury activities, reasons included head injury (50 %), polytrauma (30 %), and concern for reinjury (30 %).

**Discussion and conclusions:**

Costs of one head injury ED admission may exceed $6000, compared with $60 for one helmet. In-person provision of helmets, education, and resources is an adoptable, cost-effective intervention for improving safety awareness and reducing reinjury risk. Next steps include expanding QII implementation and refining evaluation metrics.

## Highlights

• Helmets reduce head injury severity, but only 18–22 % of U.S. adults wear them.
• We present a quality improvement initiative (QII) to improve helmet usage.
• Head injury patients were provided helmets and education in the emergency department.
• In 21 patients, 100 % reported improved safety awareness and 82 % used their helmet.
• Our QII is an adoptable, cost-effective intervention to improve safety awareness.

## 1 Introduction

It is well-established that helmets reduce traumatic brain injury (TBI) severity and mortality (Kuo et al., 2017; Persaud et al., 2012; Attewell et al., 2001). Unhelmeted patients in bicycle, scooter, and skateboard crashes with head injuries incur elevated risks of traumatic intracranial hemorrhage (Wagner et al., 2012; Allen et al., 2022), need for acute medical evaluation and hospital admission (Ganti et al., 2013), and need for neurosurgical intervention (Dagher et al., 2016) compared to their helmeted counterparts. Helmets decrease the risk of head injury in cycling crashes by 66–85 % (Thompson et al., 2000). The cost of a helmet is approximately $60 while the cost of an emergency department (ED) admission for TBI in the United States (U.S.) exceeds $6000 (Dismuke et al., 2015; National Academies of Sciences et al., 2022).

Prevention of a single head injury reduces the lifetime risk of head injuries (Saunders et al., 2009). Patients who sustain one TBI are at increased risk of sustaining subsequent TBIs, leading to further risks of death, deficits, loss of quality of life, and impact to the healthcare system (Saunders et al., 2009; Corrigan et al., 2013; Dams-O’Connor et al., 2013a; Hayward et al., 2018). Sustaining a repetitive TBI (rTBI) in the first several weeks can cause second impact syndrome, including cerebral edema, brain herniation, and death (Bey and Ostick, 2009). In the long term, a rTBI during the first year post-injury is associated with poorer recovery across functional, post-concussive, mental health, and quality of life domains (Etemad et al., 2023).

Despite widespread evidence that helmets prevent TBI and reduce its severity, 18–22 % of U.S. adults wear helmets during bicycle, scooter, and roller sport activities (Bolen et al., 1998; Rodgers, 1995). For these reasons, initiatives to improve helmet use constitute an important strategy to reduce rTBI and their related burden to patients, caregivers, and healthcare systems. Barriers to adopting helmet use include cost, wearability, and lack of education (DiGuiseppi, 1989). Several quality improvement projects, largely targeting children, have been conducted across the globe with varying degrees of success (Allabaugh et al., 2008; Falavigna et al., 2012; Koestner, 2012; Roehler et al., 2013; Ederer et al., 2016; Rivara et al., 1994; Rouzier and Alto, 1995; Lee et al., 2000; Britt et al., 1998; Lucke-Wold et al., 2020; Adams et al., 2014). Many of these initiatives successfully increased helmet use in pediatric patients, though the methods of education delivery, intervention, and assessment of helmet use (observation vs. self-report) vary.

We present a novel clinical care quality improvement initiative (QII) to improve helmet usage, education, and safety awareness in bicycle, scooter, and skateboard-related head injury patients presenting to a U.S. Level 1 trauma center. Our initiative is unique in providing in-person TBI education and resources with free helmets, with the intention to reduce rTBI. We aimed to assess the feasibility of implementing our QII and to describe the characteristics and patient perspectives of our cohort.

## 2 Methods

### 2.1 Design, setting, and participants

**Fig. 1:**
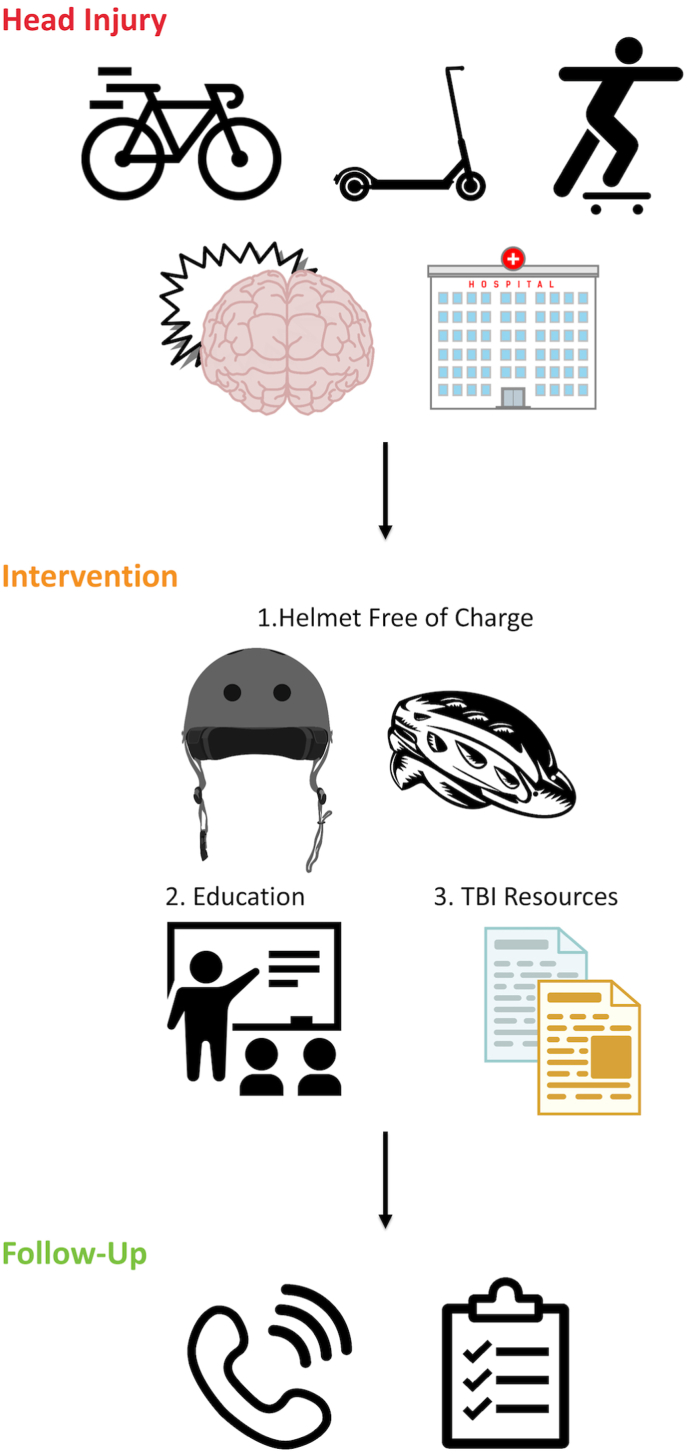
Quality Improvement Initiative Framework and Methodology **Caption:** Summary of the quality improvement initiative methodology. TBI = traumatic brain injury.

Head injury patients presenting to a single Level 1 trauma center ED after bicycle, scooter, and skateboard accidents without helmets, or with lost or damaged helmets, participated in this QII (Fig. 1). Patients were enrolled by convenience sampling from March 8th, 2023 to May 19th, 2024 as part of quality improvement efforts at the Department of Neurological Surgery at Zuckerberg San Francisco General Hospital (San Francisco, California, United States of America). Eligible patients were identified by verbal report from hospital staff or by a trained volunteer who reviewed ED admissions for 1–2 h per week. Three patients approached refused a helmet, and one patient was missed due to discharge prior to helmet receipt. In the ED, patients were provided helmets free-of-charge and in-person review of TBI education and resource packets consisting of injury statistics, publicly available TBI facts and figures, and information on post-discharge follow-up care. The resource packets contained information about TBI-specific assistive technology, cognitive rehabilitation, occupational therapy, TBI support groups, substance abuse, wellness, and mental health programs **(**Supplementary Material 1**)**. A physician, nurse, and/or departmental volunteer provided TBI education and resources to each patient.

Patients answered a departmental survey during their ED admission and at ≥1 telephone follow-up between two-weeks to one-year post-discharge to assess helmet usage, safety awareness, and rTBIs. The follow-up timeframe was selected to align with existing departmental efforts to contact patients within the first month post-discharge. All patients received a phone call within the first month, however a subset (9 of 21) completed their follow-up visit after one month due to challenges and/or conflicts in scheduling their QII appointment. Patients were contacted every 1–2 months until their QII follow-up was completed. Survey questions asked at each timepoint are summarized in Table 2. At follow-up, patients were re-provided with TBI education and resources if they were not wearing the helmet and/or had questions. A trained departmental volunteer answered patient questions regarding TBI education and community resources. Patients completed a second phone call if they requested to connect with additional medico-social resources after the first phone call.

**Table 1:** Sociodemographic and clinical characteristics.

Variable	Number of TBI Patients (N = 21)
Age	
Mean (SD)	37.7 (12.5)
**Sex**	
Male	15 (71.4 %)
Female	6 (28.6 %)
**Race/Ethnicity**	
Non-Hispanic White	9 (42.9 %)
Asian	6 (28.6 %)
Black	2 (9.52 %)
Hispanic	5 (23.8 %)
**Language**	
English	17 (19.0 %)
Spanish	4 (81.0 %)
**Prior TBI**	
No	9 (42.9 %)
Yes	11 (52.4 %)
Unknown	1 (4.76 %)
**Mechanism of Injury**	
Bicycle vs. Ground	6 (28.6 %)
Bicycle vs. Motor Vehicle	2 (9.52 %)
Scooter vs. Ground	9 (42.9 %)
Scooter vs. Motor Vehicle	1 (4.76 %)
Skateboard vs. Ground	2 (9.52 %)
Skateboard vs. Motor Vehicle	1 (4.76 %)
**Helmeted**	
No	17 (81.0 %)
Yes, but damaged or lost	4 (19.0 %)
**ED Arrival GCS**	
Median [IQR]	15 [14–15]
Mean (SD)	14.3 (1.6)
**CT Status**	
Negative	11 (52.4 %)
Positive	8 (38.1 %)
Not Ordered	2 (9.52 %)
**Highest Level of Care**	
Emergency Department	12 (57.1 %)
Hospital Ward	5 (23.8 %)
Intensive Care Unit	4 (19.0 %)

**Caption:** Sociodemographic and clinical characteristics at hospital admission for acute TBI. CT = computed tomography; GCS = Glasgow Coma Scale; IQR = interquartile range; SD = standard deviation; TBI = traumatic brain injury.

**Table 2:** Survey responses at follow-up.

Variable	Number of TBI Patients
*Baseline and/or Follow-Up (N=21)*	
Has your perspective on helmet use changed?	
Yes	21 (100 %)
No	0 (0 %)
***Follow-Up (N = 21)***	
**Have you had another head injury?^a^**	
Yes	1 (4.76 %)
No	20 (95.2 %)
**Have you resumed riding your bicycle, scooter, or skateboard? (N = 21)**	
Yes	11 (52.4 %)
No	10 (47.6 %)
**If you have not resumed riding your bicycle, scooter, or skateboard, why?^b^ (N = 10)**	
Too symptomatic or injured to resume roller sports due to TBI	5 (50.0 %)
Polytrauma	3 (30.0 %)
No longer doing roller sports out of concern for reinjury	3 (30.0 %)
**If you have resumed these activities, have you been using the helmet? (N = 11)**	
Yes	9 (81.8 %)
No	2 (18.2 %)
**If you have not been using the helmet, why? (N = 2)**	
Inconvenient	1 (50.0 %)
Feels restrictive and hot	1 (50.0 %)
**Have you informed others about the importance of wearing a helmet? (N = 21)**	
Yes	15 (71.4 %)
No	1 (4.76 %)
Not asked**^c^**	5 (23.8 %)
**Are there any resources we can connect you with? (N = 21)**	
Yes	9 (42.9 %)
No	12 (57.1 %)
**Do you have any questions about your TBI or resources? (N = 21)**	
Yes	8 (38.1 %)
No	13 (61.9 %)

**Caption:** Semi-structured survey questions and patient responses at follow-up telephone visit. **^a^** Head injury = injury to the head with loss of consciousness, posttraumatic amnesia, or alteration of consciousness. **^b^** One patient responded endorsed not using the helmet due to both polytrauma and concern for reinjury; **^c^** Question was added to interview guide later; TBI = traumatic brain injury.

**Fig. 2:**
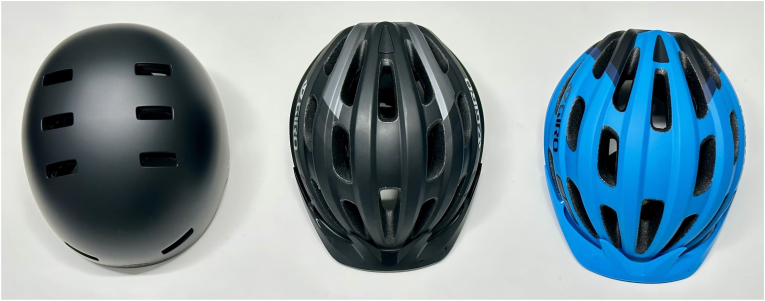
Adult and Pediatric Skateboard and Bicycle Helmets **Caption:** Photograph of helmet options in the quality improvement initiative (QII). Adult Bell skateboard helmet (left), adult Giro bicycle helmet (middle), and pediatric Giro bicycle helmet (right) are displayed.

Grant funding to purchase the helmets was obtained from the San Francisco Healthy Hearts Foundation (Community Challenge Grant Award #71933), awarded to the institution (Department of Neurological Surgery, Zuckerberg San Francisco General Hospital, San Francisco, California, United States of America). This funding was provided only for helmet purchase and did not cover staff salaries; all efforts by physicians, nurses, and QII staff were volunteer-based. Adult Bell skateboarding helmets (Bell Sports, Irvine, California, U.S.) in small, medium, and large sizes, or universal-sized Giro bicycle helmets (Giro, Scotts Valley, California, U.S.) were provided to patients depending upon their preferred size and style (Fig. 2). Giro pediatric universal-sized bicycle helmets were available for pediatric patients. All QII patients signed waivers indicating that they would use the helmet as instructed, follow manufacturer instructions, and would not sell the helmet. This analysis was conducted in accordance with the Standards for Quality Improvement Reporting Excellence (SQuIRE) reporting guidelines (Ogrinc et al., 2016).

## 3 Ethics statement and approval

The University of California, San Francisco Institutional Review Board (IRB) reviewed this study and classified it as exempt from full IRB review (Study # 24-42961). The IRB approved a waiver of informed consent because data contained herein was collected for quality improvement within the Department of Neurological Surgery at Zuckerberg San Francisco General Hospital (San Francisco, California, United States of America). Data were de-identified, extracted, and analyzed for the current report.

### 3.1 Sociodemographic and injury variables

Sociodemographic variables and acute injury characteristics collected during the departmental QII were analyzed in the current analysis. Sociodemographic variables included age, sex, race, ethnicity, primary language, and prior TBI history. Prior TBI was collected using the Ohio State TBI Identification Method (OSU-TBI-ID) (Corrigan and Bogner, 2007; Bogner and Corrigan, 2009), which defined TBI as a head injury with loss of consciousness, post-traumatic amnesia, and/or alteration of consciousness. Acute injury variables included mechanism of injury, Glasgow Coma Scale score (GCS) (Teasdale and Jennett, 1974) at ED arrival, highest level of care, and presence of traumatic intracranial hemorrhage on head computed tomography (CT). For mechanism of injury, motor vehicles were defined as road motor vehicles (automobiles, vans, trucks, motorcycles) in our QII. Head CTs were coded as positive or negative for intracranial hemorrhage based on the attending radiologist's final read. These variables were selected to align with the National Institute of Neurological Disorders and Stroke TBI Common Data Elements routinely collected in our department for quality improvement (Maas et al., 2010; Duhaime et al., 2010).

### 3.2 Statistical analysis

Descriptive statistics were reported for quantitative analyses of characteristics and follow-up data. Means and standard deviations (SD) were used to report parametric data, and medians and interquartile ranges (IQR) for non-parametric data. Microsoft Excel was used to manage data records and statistical analyses.

## 4 Results

### 4.1 Baseline characteristics

Of 21 TBI patients in our QII, 9 (42.9 %) were White/Non-Hispanic, 6 (28.6 %) were Asian, 5 (23.8 %) were Hispanic, and 2 (9.5 %) were Black (Table 1). Seventeen patients (81 %) spoke English and 19 % spoke Spanish as their primary language. Twenty patients were adults and one was 14-years-old. Mean age was 37.7 ± 12.5 years, 71.4 % were male, and 52.4 % reported prior TBI history (Table 1).

### 4.2 Acute injury characteristics

Mechanisms of injury included scooter vs. ground (9/21, 42.9 %), bicycle vs. ground (6/21, 28.6 %), bicycle vs. motor vehicle (2/21, 9.5 %), skateboard vs. ground (2/21, 9.5 %), skateboard vs. motor vehicle (1/21, 4.8 %), and scooter vs. motor vehicle (1/21, 4.8 %) (Table 1). Seventeen patients (81 %) were unhelmeted during initial injury, while 4 (19 %) patients’ helmets were damaged or lost in the crash. The majority of scooters were electric (N = 9/10). Eleven patients (52.4 %) had negative head CT for intracranial hemorrhage, 8 (38.1 %) had positive head CT, and 2 (9.5 %) did not receive CT. Median ED arrival GCS was 15 (IQR = 14–15) (Table 1). For level of care 12 (57.1 %) were discharged from ED, 5 (23.8 %) were admitted to hospital ward, and 4 (19.0 %) were admitted to intensive care unit (ICU) (Table 1). Patients reported the following reasons for not wearing a helmet during their injury: did not have helmet (N = 3), forgot to wear helmet (N = 3), cultural (N = 2), helmet stolen (N = 1), helmet not stylish (N = 1), only wears helmets at night (N = 1), took too long to put helmet on (N = 1), or unknown reasons (N = 5).

### 4.3 Survey responses at follow-up

**Fig. 3:**
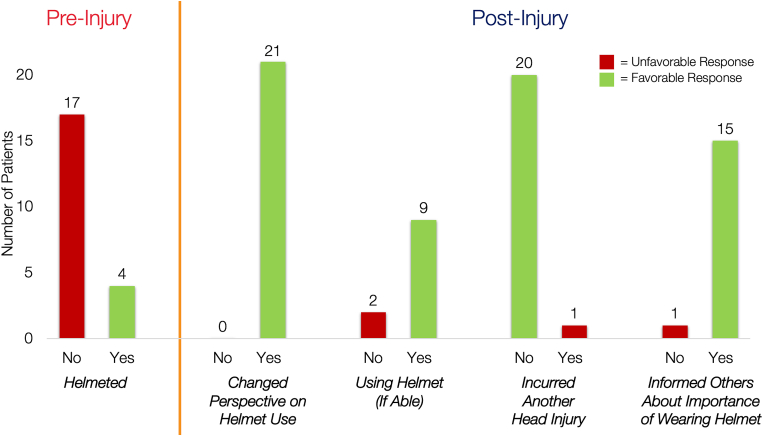
Patient Perspectives on Helmet Use Pre- and Post-Injury **Caption:** Visual representation of patient responses to interview questions at baseline (pre-injury) and follow-up (post-injury) regarding their helmet use. “Yes” and “No” represent patient responses to each interview question. Favorable responses are shown in green and unfavorable responses in red. Of note, the sample sizes are lower for “Using Helmet (If Able)” because 10 of 21 patients had not resumed their pre-injury bicycle, scooter, or skateboard activities at follow-up, and for “Informed Others About Importance of Wearing Helmet” because this interview question was added to the study after 5 patients had already completed follow-up.

All patients reported changed perspectives on helmet use during QII participation (Fig. 3), which were described as positive and helpful for improving safety awareness and head injury protection during their activities. All patients completed at least one follow-up visit. Median number of days from injury to first follow-up was 18 (IQR = 14–88). Two patients completed a second phone call between 93 and 96 days. One patient did not use their helmet at their first follow-up visit due to polytrauma, but reported wearing the helmet at subsequent follow-up.

Ten of 21 patients (47.6 %) did not resume their pre-injury vehicular and/or roller sports activities post-injury (Table 2). Reasons included head injury (5/10, 50 %), polytrauma (3/10, 30 %), and concern for reinjury (3/10, 30 %), with one patient endorsing both polytrauma and concern for reinjury. Of the 11 patients able to resume their pre-injury bicycle, scooter, and/or skateboard activities, the majority (9/11, 81.8 %) were wearing their helmet at follow-up (Fig. 3). One patient did not wear the helmet due to inconvenience, and one because the helmet felt restrictive and hot. Ninety-five percent did not have rTBI after their TBI of enrollment within the timeframe assessed (Table 2).

At follow-up, patients were re-provided with TBI education and resources as needed. Forty-three percent requested to be connected with additional post-discharge TBI resources during follow-up. Community resources requested included post-acute follow-up care (N = 3), physical therapy (N = 2), mental health (N = 1), health insurance (N = 1), and general TBI resources (N = 3). Additionally, 8 patients (38.1 %) had questions for the QII team about their TBI and/or resources provided. Regarding community awareness, 71 % endorsed informing other people about the importance of wearing a helmet (Fig. 3).

## 5 Discussion

### 5.1 Implementation and feasibility

Our study found that it was feasible to execute our project and provide education, helmets, and resources to 21 patients with bicycle, scooter, or skateboard-related head injuries during their ED and/or hospital stay. All patients endorsed an improved understanding of risk reduction strategies and helmet use after undergoing our QII. Importantly, 95 % did not have a repeat head injury and, of patients able to resume roller sports, 82 % were wearing a helmet. Our study uniquely focused on primarily adult trauma patients at a U.S. Level 1 trauma center, provided in-person TBI education and resources in conjunction with new helmets, and aimed to reduce rTBI. Our analysis demonstrated that our QII framework was feasible to implement and will inform the design of future QIIs.

Polytrauma and TBI severity represented barriers to assessing helmet usage in our cohort: 48 % of patients could not use the helmet because they were unable to resume biking, skateboarding, and scootering due to their TBI and polytrauma. Polytrauma is a known confounder of TBI outcomes (Yue et al., 2020a). Additionally, the majority of TBI recovery occurs between 3 and 6 months post-injury (Schretlen and Shapiro, 2003; Carroll et al., 2004; McCrea et al., 2009). As such, future QIIs should consider assessing helmet usage at 3 and/or 6 months post-injury to reduce these confounders.

Notably, 81 % of patients in our cohort did not wear a helmet at baseline, and factors attributed to non-helmet use included cost, forgetting to wear a helmet, concerns for theft, fashion, and cultural attitudes. After undergoing our QII, two patients reported not wearing a helmet at follow-up due to inconvenience and fit. Several studies have identified factors that contribute to non-helmet use consistent with our results (Finnoff et al., 2001; Ferraro et al., 2018; Piotrowski et al., 2020) and note a role for normative beliefs (Ledesma et al., 2019). To minimize style concerns and poor fit, patients selected one of two helmet styles and we offered universal-sized helmets. Future QIIs may consider implementing universal-sized skateboard helmets and reviewing a “frequently asked questions” list with patients to address helmet use barriers.

### 5.2 Repetitive TBI

In our cohort, 52 % of patients reported a history of prior TBI. Past studies estimate prior TBI incidence to be 14–23 % in adults (Corrigan et al., 2013; Dams-O’Connor et al., 2013a; Dams-O’Connor et al., 2013b). The risk of subsequent TBI(s) can double or triple with each TBI incurred (Saunders et al., 2009). Our QII cohort, characterized by a high rate of prior TBI and unhelmeted status, may represent a vulnerable sample at risk for additional TBI(s). In our QII, one patient (5 %) sustained a repeat, helmeted head injury. Other studies report a rate of 3–12 % for recurrent TBI, depending upon the timeframe and method to track TBIs (Saunders et al., 2009; Etemad et al., 2023; Winqvist et al., 2008; Bannon et al., 2021; Theadom et al., 2015). Clinicians may consider TBI history and helmet use status to identify patients at risk for repetitive neurotrauma and provide TBI counseling.

### 5.3 Follow-up care and resources

Our QII provided all patients with TBI resource packets at discharge, which included community resources spanning multimedia TBI education, post-acute follow-up care, assistive technology, and other community resource domains. Prior literature suggests that 42 % of TBI patients receive educational material at discharge and 44 % see a clinician within 3 months post-discharge (Seabury et al., 2018). In our cohort, the most commonly requested resource was post-acute follow-up care information, underscoring the importance of providing this resource to patients. In addition, our QII provided resources at multiple timepoints, with 43 % of QII patients requesting additional resources at follow-up. Delivery of education and resources in an acute emergency setting is challenging, and additional provision at follow-up may benefit patients. Additionally, building a system of post-acute TBI follow-up care may support TBI patients in their recovery (National Academies of Sciences et al., 2022; Eagle et al., 2025) and decrease the likelihood of reinjury.

### 5.4 Public health implications and future directions

In the U.S., there are no state or federal laws requiring adults to wear helmets for bicycle, electric scooter, skateboard, and other roller sport activities, though some local mandates exist. Many states require minors aged <18 years to wear bicycle helmets, with most evidence suggesting that such laws reduce head injuries and fatalities (Du et al., 2020). Our study noted a high number of unhelmeted electric scooter riders (N = 8), which is legal in California (Codes: Cross) and consistent with current literature noting an increase in these injuries (Namiri et al., 2020), suggesting targeted public health efforts to increase helmet use may benefit this cohort. QIIs can promote helmet use, in addition to legislation and other local initiatives (Yue et al., 2020b, 2020c). Several QIIs have been conducted in the U.S. and globally to reduce TBI with the majority successfully increasing helmet use (Allabaugh et al., 2008; Falavigna et al., 2012; Koestner, 2012; Roehler et al., 2013; Ederer et al., 2016; Rivara et al., 1994; Rouzier and Alto, 1995; Lee et al., 2000; Britt et al., 1998; Lucke-Wold et al., 2020; Adams et al., 2014). Thoughtful consideration of TBI education delivery and how to objectively track helmet use is important. Further prospective studies in larger samples are needed to validate the feasibility and efficacy of helmet QIIs.

### 5.5 Limitations

A limitation of this study was utilizing patient self-report to track helmet use. Additional methods for tracking helmet use, such as direct observation and other objective data collection methods, should be implemented in future QIIs. Although our primary aim was feasibility, our study was limited by a small sample size, and validation of our QII framework is needed in larger cohorts.

## 6 Conclusions

In-person provision of helmets, TBI education, and resources after bicycle, scooter, and skateboard-related head injury is a readily adoptable and cost-effective intervention for the reduction of future injuries in the U.S. trauma center ED setting, in contrast with the high costs of a single TBI to patient economic self-sufficiency and healthcare systems. All patients reported improvements in safety awareness after undergoing our QII. Our QII framework awaits validation in larger cohorts with continued expansion and refinement of QII metrics and outcome assessments.

## Data sharing statement

Deidentified supporting data for the study herein can be made available upon reasonable request to the corresponding author.

## Funding

This study, including data collection, analyses, and reporting, was unfunded. The helmets used as part of this quality improvement initiative were already available for departmental use funded by the San Francisco Healthy Hearts Foundation (Community Challenge Grant Award #71933), awarded to the institution (Department of Neurological Surgery, Zuckerberg San Francisco General Hospital, San Francisco, California, United States of America).

## Declaration of competing interest

The authors declare the following financial interests/personal relationships which may be considered as potential competing interests: This study received funding to purchase helmets from the San Francisco Healthy Hearts Foundation (Community Challenge Grant Award #71933), awarded to the institution (Zuckerberg San Francisco General Hospital). Data collection and analysis efforts for this study were unfunded and volunteer-based. If there are other authors, they declare that they have no known competing financial interests or personal relationships that could have appeared to influence the work reported in this paper.
