# Supplementary material for: Risk reduction after bicycle, scooter, and skateboard-related head injuries through helmet use and brain injury education: A trauma center quality improvement initiative
Source: Brain Spine. 2025 Oct 28;5:105858. doi: 10.1016/j.bas.2025.105858 (PMC12607080; doi:10.1016/j.bas.2025.105858)
Supplement: Multimedia component 1 [file mmc1.pdf]

What is a traumatic brain injury (TBI)?

What happens in a TBI?

- A blow to the head can occur in many ways, such as in a car accident, fall, bicycle accident, assault, and sports injury.
- Sometimes the skull can break or the brain can be shaken around inside the skull, which can cause bruises to the brain or *hematomas.*
- The brain can swell after an injury, which can take up to six months to go down.
- Depending on the injury, a TBI can cause changes in thinking, smelling, seeing, talking, feelings, and how you move your body.
- **Re-injury** to the brain during recovery from brain injury can be fatal.

Common Causes of TBI

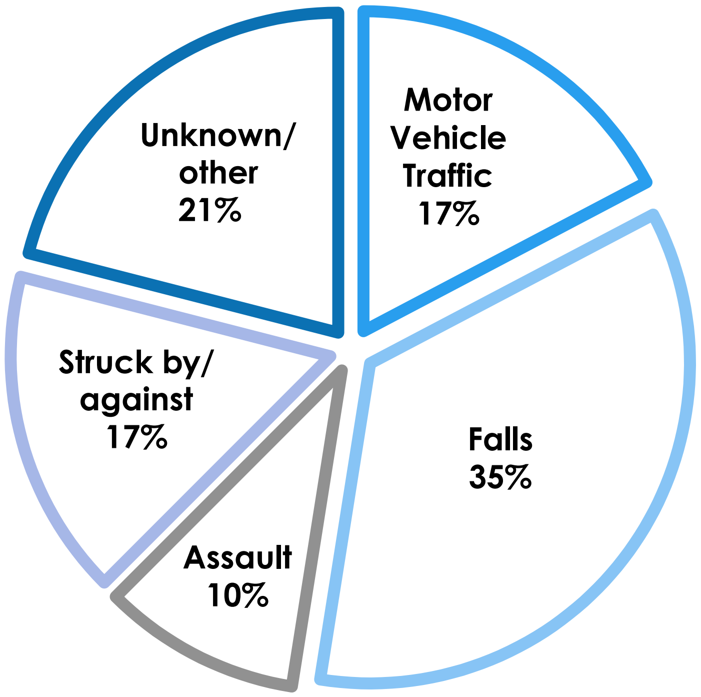

How will I feel? What can I do about it?

Symptoms you may experience:

- You may experience a range of symptoms including poor attention/concentration, trouble thinking, altered judgment, forgetfulness, repeating yourself, trouble finding your words, slowed thinking, and difficulty with planning, and organization
- Problems with falling sleep, sleeping too much or little, running out of energy more quickly, headaches, sensitivity to light or noise, ringing in ears, blurred vision, dizziness, difficulty with balance, and poor sense of taste and smell
- Emotional and mood problems such as irritability, sadness, depression, nervousness, and anxiety

What can you do?

- **Pace yourself.** Take frequent breaks and naps and avoid tasks that need a lot of physical and mental energy. You may need to take time off work or school until you feel better. If naps interfere with nighttime sleep, try limiting how long you nap. You can also try to take naps earlier in the day.
- **Avoid alcohol and drugs as it can make symptoms worse and prolong recovery time.** Limit caffeine to no more than two cups of coffee, tea, or cola.
- Know that it is okay to ask for help, and share your feelings with others. Remember that recovery is different for everyone and that it takes time.
- **Do one thing at a time**, limit noise and distractions, don't be hard on yourself, write important things down, and use a calendar to help you remember appointments and important dates.
- Speak with your hospital staff about any questions and concerns you may have.
- Try to go to sleep at the same time every night. Do relaxing things before bedtime, such as a warm bath/shower. Don't watch TV or use the computer right before you go to sleep.
- Take pain relievers for headaches as directed by your doctor. Take prescribed and over the counter medications as directed by your doctor.
- Try to do things that make you happy and reduce stress.
- Eat healthy meals and exercise regularly. Stop exercising if your symptoms, such as headache or dizziness, get worse.

When will I get better?

- Most people with mild TBIs fully recover in the weeks and months after being injured. Most symptoms go away on their own with little treatment
- Most people with moderate to severe TBIs continue to experience symptoms and may need help returning to activities.
- The greatest amount of neurological recovery occurs in the first 6 months after your injury and it can continue for up to 2 years. After this, TBI survivors can still recover as they learn to work around their difficulties.
- The **Rancho Level of Cognitive Functioning** provides a good description of what to expect with cognitive recovery. You can view a description at this website: https://file.lacounty.gov/SDSInter/dhs/218115_RLOCFOriginalFamilyGuide-English.pdf

If your cognitive, mood, sleep, or other symptoms do not improve within several weeks to months post-injury, seeking care from a neuropsychologist, rehabilitation provider, speech therapist, sleep specialist, or psychotherapist may be beneficial.

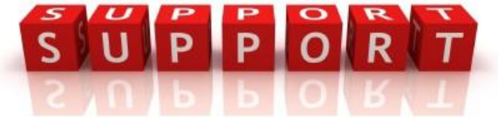

Free Support & Education Group:

Bring your family and friends!

When: 1st and 3rd Thursday every month 3:30-4:45pm

Where: Virtual by Zoom, contact Michelle Diaz (michele.diaz1@sfdph.org, 415-996-0738) for Zoom meeting information

Neurosurgery Patient Helpline: 415-206-4093

What do I need to watch out for?
How can I prevent this from happening again?

Call 911 or go to the nearest hospital if you:

- Have a **severe headache** and it does not go away after taking medication
- Are **vomiting (throwing up**) and cannot stop, and/or cannot keep your balance
- Have a **seizure**, and/or have **clear liquid or blood** coming from your ears or nose
- **Cannot stay awake** or wake up a loved one
- Are suddenly **unable to move body parts, see, and talk**

Safety:

- **ALWAYS** wear a seatbelt in a car
- **ALWAYS** wear a helmet while on a bicycle, motorcycle, contact sports, skating, skateboarding
- Use proper child safety seats
- **AVOID alcohol and drugs**
- **NEVER** drive under the influence of alcohol or drugs

Falls Prevention:

- Use the handrails for safety
- Use enough lighting
- Place bars on windows to prevent someone from falling
- Sit on safe stools and chairs
- Do not place items in walkways

Firearm Safety:

- Store guns unloaded and keep in a locked cabinet
- Store ammunition separate from guns

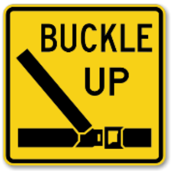

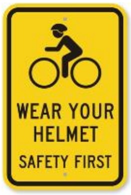

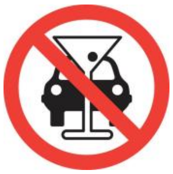
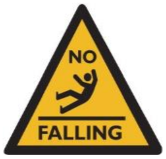

Alcohol: How much is too much?

EACH OF THESE IS A STANDARD DRINK

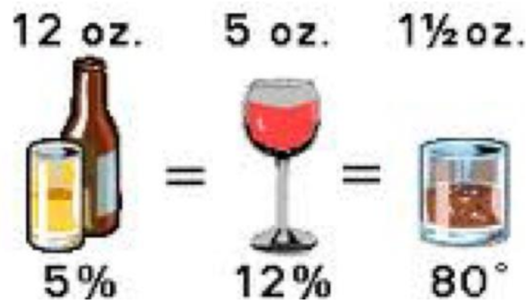

HOW MUCH IS TOO MUCH?

Drinks	Per Week	Per Occasion
Men	14	4
Women	7	3
>65 yrs	7	3

COULD YOU HAVE A PROBLEM WITH ALCOHOL?

- Have you ever felt you should ***cut down*** on your drinking?
- Have people ***annoyed*** you by criticizing your drinking?
- Have you ever felt bad or ***guilty*** about your drinking?
- ***Eye opener***: Have you ever had a drink first thing in the morning to steady your nerves or to get rid of a hangover?

If you answered ***YES*** *to two or more of these questions, you may have a problem with alcohol.*

FREE HELP IS AV AILABLE
FOR
DRINKING AND DRUGGING

(See Below)

Alcohol and Drug Treatment

TREATMENT ACCESS PROGRAM

- Provides information and referrals to various treatment programs in San Francisco
  - 1380 Howard Street, Ste. 1000
  - 415-255-3737
  - 888-246-3333
  - 800-750-2727
  - tapsf@sfdph.org

FAMILY SUPPORT

- Al-Anon: 415-834-9940
  - Spanish: 650-794-9654
  - www.al-anon.org

ANONYMOUS TREATMENT

AA: 415-674-7821

- Spanish: 415-824-1834
- Hard of hearing: dial 711, ask for 415-674-1821
- www.aasf.org

Narcotics Anonymous

- 415-621-8600
- www.sfna.org

Cocaine Anonymous

- 415-821-6155
- www.norcalca.com

Internet Based Support

For those who prefer to seek help via internet using online meetings, activities, and face-to-face meetings. The services are free.

www.lifering.org
www.sossobriety.org
www.womenforsobriety.org

www.moderation.org
www.smartrecovery.org
www.facesandvoicesofrecovery. org

Follow Up Care and Support

TBI MEDICAL SERVICES:

San Francisco TBI Network
Janet Pomeroy Center
207 Skyline Blvd.
San Francisco, CA 94132
415-665-4100 ext. 1782
www.janetpomeroy.org

Brain & Spinal Injury Center at University of California, San Francisco General Hospital & Trauma Center
1001 Potrero Ave. Bldg. #1, Room 101
San Francisco CA, 94110
415-206-8300
www.brainandspinalinjury.org

Brain Injury Network of the Bay Area
1132 Magnolia Ave.
Larkspur, CA 94939
415-461-6771
www.mbin.org

OTHER RESOURCES:

Brain Injury Association of America
800-444-6443
www.biausa.org

Traumatic Brain Injury Resource Directory
www.tbi-sci.org/tbird

Seizure Tracker
www.seizuretracker.com

TBI Survival Guide
www.tbiguide.com

RECOVERY & CAREGIVER RESOURCES:

SFGH Trauma Recovery Center
2727 Mariposa St. Suite 100
San Francisco, CA 94110
415-437-3000
www.traumarecoverycenter.org

Family Caregiver Alliance
690 Market St. Suite 600
San Francisco, CA 94104
415-434-3388
www.caregiver.org

Brain Trauma Foundation
212-772-0608
www.braintrauma.org

TBI Skills Group: John Adams Community College
415-561-1005

FINANCIAL SERVICES:

Victim Compensation Fund
800-777-9229
www.boc.ca.gov

Victim Witness Assistance Center
850 Bryan St. Room 320
San Francisco, CA 94103
415-553-9044

State Disability Insurance (SDI)
1-800-480-3287

Employment Development Dept.
1-800-300-5616

Bay Area Legal Aid
415-354-6360

Traumatic Brain Injury Resources

Websites/TBI Education

TBI Survival Guide	www.tbiguide.com
TBI Model Systems Knowledge Translation Center<br>Keep Tabs on Seizures	https://msktc.org/tbi<br>www.seizuretracker.com
Traumatic Brain Injury Resource Directory	https://brainandspinalinjury.org/wp-content/uploads/2018/09/Traumatic-Brain-Injury-Resource-Directory-TBIRD-VI.pdf
Brain Injury Association of America	www.biausa.org
Brain and Spinal Injury Center	https://brainandspinalinjury.org/patients-families/traumatic-brain-injury/
CDC	https://www.cdc.gov/traumaticbraininjury/mtbi_guideline.html
UCSF Department of Neurosurgery	http://neurosurgery.ucsf.edu
Brain Trauma Foundation	www.braintrauma.org
ARCH National Respite Network	Joy Scott, Co-President<br>530-755-3500<br>https://archrespite.org/
Brain Line	https://www.brainline.org/
Concussion Alliance	https://www.concussionalliance.org/about-concussions

Wellness/Media

Love Your Brain Non-profit Organization	Group discussion and yoga for TBI patients and caregivers.<br>www.loveyourbrain.com
ZSFG Community Wellness Center	628-206-4995<br>http://sfghwellness.org
Meditation Phone Apps	Insight Timer<br>Headspace
"The Crash Reel"	2013 documentary
"The Rider"	2017 film
The Water Giver	2010 book
The Brains Way of Healing	2016 book

BRAIN AND SPINAL INJURY CENTER
San Francisco General Hospital
University of California at San Francisco

ZUCKERBERG SAN FRANCISCO GENERAL
Hospital and Trauma Center

Lost in My Mind: Recovering from a TBI	2014 memoir
Crash Course: A Self-Healing Guide	2016 guide
Broken: Living with a Brain Injury	YouTube series (Part 1-5)

TBI Medical Services/Follow-Up

UCSF Neurorecovery Clinic	1651 Fourth St., Suite 232, San Francisco, CA 94158<br>415-514-1252<br>https://www.ucsfhealth.org/clinics/neurorecovery-clinic
San Francisco TBI Network Resources through the Shurig Center for Brain Injury Recovery	1132 Magnolia Ave., Larkspur, CA 94939<br>415-461-6771<br>https://schurigcenter.org/information-resources/resources-directory/?cn-s=&cn-cat=0
ZSFG Neurosurgery Clinic	4M Specialty Care Clinic, 1001 Potrero Ave, Building 5, Floor 4, Room 4M, San Francisco, CA 94110<br>628-206-4420<br>https://zuckerbergsanfranciscogeneral.org/es-us/location/neurosurgery/
UCSF Sports Concussion Center	Locations in San Francisco, Oakland, Walnut Creek, and Marin<br>415-353-1915 (SF), 510-601-3900 (East Bay)<br>https://sportsconcussion.ucsf.edu/
California Concussion Institute	900 Lafayette St., Suite 105<br>Santa Clara, CA 95050<br>(408) 625-7101<br>https://westcoastdocs.com/concussion-care-2/
Brain and Spinal Injury Center, Zuckerburg San Francisco General Hospital & Trauma Center	2540 23rd St., Bldg. #7, Rm 5212, San Francisco, CA 94110<br>415-206-8300<br>www.brainandspinalinjury.org

Coping/Cognitive Rehab/Occupational Therapy/Rehabilitation

Coping with Acquired Brain Injury Course at John Adams Campus of San Francisco City College	Joyce Foreman<br>415-561-1005
Janet Pomeroy Center Brainstorm Social, Recreation, and Integration Program	207 Skyline Blvd., San Francisco, CA 94132<br>415-665-411 ext. 1782<br>https://www.prrcsf.org/tbi-abi

	www.janetpomeroy.org
Shurig Center for Brain Injury Recovery	1132 Magnolia Ave, Larkspur CA 94939<br>415-461-6771<br>https://schurigcenter.org/
Centre for Neuro Skills	2200 Powell, Suite 600, Emeryville, CA 94608<br>1-800-922-4994 | 510-318-8600<br>https://www.neuroskills.com/locations/san-francisco/
On Track Program: Mr. Diablo Unified School District Day/Education program for adults with acquired brain injury	1266 San Carlos Ave.<br>Concord, CA 94518<br>925-685-7340 ext. 6721<br>https://mdae-mdusd-ca.schoolloop.com/awd
ZSFG 6-week Yoga & Meditation Program	Michele Diaz, RN, MS<br>628-206-2906
Bay Area Physiatrists	Dr. Scott Rome, CMPC/Sutter Health, 415-523-6555<br>Dr. Lisa Pascual, UCSF Health, 628-206-8811
Stanford Neuroscience Outpatient Neurologic Rehabilitation Program (Occupational, physical, and speech-language therapy)	213 Quarry Rd, Palo Alto, CA 94304<br>Phone: 650-498-3333<br>https://stanfordhealthcare.org/medical-clinics/outpatient-neurologic-rehabilitation-program.html
Rehab Without Walls<br>(Residential, outpatient, and home services: physical, occupational, and speech-language therapy)	1038 Leigh Avenue, Suite 101A, San Jose, CA 95126<br>Phone: (408) 559-9020<br>https://www.rehabwithoutwalls.com/

Assistive Technology and Reading Resources

Sensus Access	Web application that converts PDFs to e-books.<br>https://www.sensusaccess.com/convert-a-file/
Mac Accessibility	Mac OS X and iOS has built-in accessibility tools for iPhone, iPad, and Mac such as Mac text-to speech, iPhone screen reader tool, and reducing screen brightness.<br>https://www.apple.com/accessibility/<br>https://support.apple.com/guide/mac-help/have-your-mac-speak-text-thats-on-the-screen-mh27448/mac
Windows 10 Accessibility	Windows 10 has similar accessibility features and a

	built-in screen-reading app called Narrator. https://www.microsoft.com/en-us/windows/accessibility-features?activetab=pivot_1%3aprimaryr2&r=1 https://support.microsoft.com/en-us/windows/complete-guide-to-narrator-e4397a0d-ef4f-b386-d8ae-c172f109bdb1
Natural Reader	Online text to speech web application with high quality voices. https://www.naturalreaders.com/software.html
Balabolka	Text to speech program with free download for windows 10. http://www.cross-plus-a.com/balabolka.htm
F.lux	An application that decreases computer screen brightness and may assist with light sensitivity. https://justgetflux.com/

Mental Health Support

San Francisco Warm Lines: Free on-demand mental health support	Mental Health Triage Warm Line: 1-855-845-7415<br>Warm line chat online: mentalhealthsf.org Mental Health Access Helpline: 415-255-3737
Suicide Prevention Hotline	National Hotlines: 988 (24/7)<br>San Francisco Suicide Prevention Hotline: 415-781-0500<br>Crisis Text-741741

Support Groups

ZSFG TBI Support Group	Carol Blecker, LCSW<br>415-437-3008<br>Michele Diaz RN, MS<br>415-996-0738<br>Jary Larsen, PhD<br>628-206-8698
Family Caregiver Alliance (English and Spanish/Patient and caregiver)	415-434-3388<br>www.caregiver.org/support-groups
Schurig Center for Brain Injury Recovery (Patient and Caregiver groups)	415-461-6771<br>www.schurigcenter.org
Brain injury Survivors Deserve Better Support Group	John Hatten<br>415-601-9654

East Bay Brain Injury Support Group	Dr. Pamela Paradowski<br>510-433-9955
Brainstorm at the Pomeroy<br>Recreation & Rehabilitation Center	Maridine Zieman, Director<br>415-213-8520<br>mzieman@prrcsf.org
Brain Injury Peer Support Group at the Stanford Synapse Brain Injury Support Group	650-319-6110<br>www.synapsbi.stanford.edu
Braintrust at Berkeley	510-550-5897<br>braintrustatberkely@gmail.com

Case Management

SFGH Trauma Recovery Center	415-437-3000<br>www.traumarecoverycenter.org
Positive Resource Center	415-777-0333<br>www.prcsf.org
Independent Living Resource Center	415-543-6222 ext. 1100<br>www.ilrcsf.org
Bay Area Legal Aid	415-354-6360<br>www.baylegal.org
Tenderloin Health	415-674-6140<br>www.healthright360.org

Disability

Independent Living Skills Resource Center of SF	www.ilrcsf.org<br>415-543-6222 ext. 1100
City and County of San Francisco Human Services Agency Adult Day Programs	https://www.sfhsa.org/services/disability-aging-services/community-activities/adult-day-programs415-557-5000
Bay Area Outreach and Recreation Program (BORP)	www.borp.org<br>510-849-4663
Paratransit (transportation)	415-351-7050
Disability Rights California<br>Protection & advocacy for individuals with traumatic brain injury program (PATBI)	1-800-776-5746 (Voice)<br>1-800-719-5798 (TTY)<br>https://www. Disabilityrightsca.org<br>https://www.disabilityrightsca.org/publications/protection-advocacy-for-individuals-with-traumatic-brain-injury-patbi-program

Benefits Assistance/Financial Services/Job Assistance

State Disability Insurance (SDI)	1-800-480-3287
Employment Development Department	1-800-300-5616
Positive Resource Center	1-415-777-0333
Department of Rehabilitation (DOR)	Theresa Woo<br>415-600-4875<br>https://www.dor.ca.gov/
Free San Francisco Help Charts	www.FreePrintShop.org
PEP Jobs	Tom Post<br>415-600-4875<br>postt@sutterhealth.org

County Adult Assistance Program (CAAP): General Assistance (income)	415-558-2227 https://www.sfhsa.org/services/jobs-money/county- adult-assistance-programs-caap

Skilled Nursing Facilities

San Francisco Campus for Jewish Living	415-334-2500
Lawton Healthcare Center	415-566-1200
Kindred Transitional Care and Rehabilitation-Tunnell-Currently operated by Generations Healthcare	415-673-8405 http://lifegen.net/tunnell/index.html
Pacific Heights Transitional Care Center	415-563-7600 http://phhealthsf.com/
The Avenues Transitional Care Center	415-661-8787
SF Nursing Center	877-252-4469
St. Francis Convalescent Pavilion	650-994-3200
San Bruno Skilled Nursing Hospital	650-583-7768
Burlingame Long-Term Care Center	650-692-3758

Housing Assistance

San Francisco Housing Authority (SFHA)	415-241-1017
Tenderloin Neighborhood Development Corporation	415-776-2151 www.tndc.org
Episcopal Community Services SF/Next Door Shelter	https://ecs-sf.org 415-487-3300
Tenderloin Housing Clinic	https://www.thclinic.org/ 415-885-3286
Community Care Licensing Division	www.ccld.ca.gov 916-651-8848

Victim Services

California Victim Compensation Board	800-777-9229 www.boc.ca.gov https://victims.ca.gov/victims/
Victim Services Division	415-553-9044
SFGH Trauma Recovery Center	415-437-3000 www.traumarecoverycenter.org
Bay Area Legal Aid	415-354-6360 https://baylegal.org

Walk San Francisco<br>Families for Safe Streets	Aly Geller<br>415-431-9255 ext. 4<br>https://walksf.org/families-for-safe-streets/
